# Sol-Gel Combustion-Assisted Electrostatic Spray Deposition for Durable Solid Oxide Fuel Cell Cathodes

**DOI:** 10.3389/fchem.2022.873758

**Published:** 2022-04-11

**Authors:** Jongseo Lee, Sehee Bang, Wonyoung Lee

**Affiliations:** ^1^ Advanced Defense Science and Technology Research Institute, Agency for Defense Development, Daejeon, South Korea; ^2^ School of Mechanical Engineering, Sungkyunkwan University, Suwon, South Korea

**Keywords:** solid oxide fuel cell, electrostatic spray deposition, sol-gel combustion, perovskite oxide, sr segregation

## Abstract

The chemical instability of perovskite oxides containing Sr is a critical issue for the long-term operation of solid oxide fuel cells. In this study, we demonstrate a remarkable improvement in the chemical and electrochemical stability of a heterostructured La_0.6_Sr_0.4_CoO_3-δ_ (LSC)-Ce_0.9_Gd_0.1_O_1.95_ (GDC) electrode. Electrostatic spray deposition was employed to fabricate heterostructured nanoparticles in a single step with a coaxial nozzle supplying the LSC powders in the core nozzle and the GDC precursors in the shell nozzle. Moreover, the reducing fuel added to the GDC precursor solution induced the sol-gel combustion reaction in the droplet to form a uniform nanocrystalline GDC coating with high surface coverage. The high surface coverage of GDC on the LSC more significantly improved long-term stability compared with than of the bare LSC cathode at a constant current density of 1 A/cm^2^ at 600°C for 100 h.

## Introduction

Perovskite oxides containing Sr as an A-site dopant have been considered promising cathode materials because of their high catalytic activity and low activation energy (E_a_) for the oxygen reduction reaction (ORR) of solid oxide fuel cells (SOFCs), particularly those operating in the intermediate temperature regime (500–700°C) (Kawada et al., 2002). However, the overall electrochemical performance of perovskite oxide-based cathodes has been reported to deteriorate significantly during long-term operation, because of their chemical instability (Neagu et al., 2013; Myung et al., 2016; Koo et al., 2018; Choi et al., 2021). Previous research has revealed that the performance degradation originates primarily from Sr segregation toward the surface and subsequent changes in the chemistry and structure near the surface (Cai et al., 2012; Chen et al., 2012; Lee et al., 2013). Therefore, extensive research has been conducted to effectively prevent Sr segregation and develop durable SOFCs for widespread applications.

A heterostructured cathode, which contains a chemically stable layer at the surface, has been demonstrated as a promising strategy to suppress Sr segregation (Lynch et al., 2011; Ding et al., 2014; Lee et al., 2014; Chen et al., 2017; Wen et al., 2018; Choi et al., 2020a; Choi et al., 2020b; Choi and Lee, 2021). The electrostatic interactions between charged defects in perovskite oxides are one of the most dominant forces for Sr segregation, which originates from the heterogeneity in the concentration and distribution of the charged defects (Lee et al., 2010; Hamada et al., 2011; Jalili et al., 2011; Choi et al., 2020a). In particular, the enriched oxygen vacancies ( $V_{O}^{\cdot \cdot }$ , an effective charge of +2) at the surface can attract divalent dopants $\left(Sr_{A-site\;host}^{'}\right)$ by Coulombic force, owing to the lower formation enthalpy at the surface than that in the bulk, resulting in Sr enrichment at the surface. Subsequent Sr segregation beyond the solubility limit of the A-site dopants in the lattice leads to the formation of insulating Sr-rich phases at the surface, destroying the desired stoichiometry (Choi et al., 2020a). In the heterostructured cathode, Sr segregation can be suppressed by decreasing the concentration of oxygen vacancies at the cathode surface *via* defect transfer near the interfaces (Chen et al., 2018; Choi et al., 2020a). Choi et al. recently reported precise control of the concentration of oxygen vacancies in La_0.6_Sr_0.4_CoO_3-δ_ (LSC)-Ce_0.9_Gd_0.1_O_1.95_ (GDC) thin-film cathodes by exploiting defect transfer, demonstrating significantly improved stability over long-term operation (Choi and Lee, 2021). However, a thin, conformal coating on the three-dimensional porous cathodes that maximizes stability enhancement and minimizes performance degradation is challenging to fabricate. Vacuum-based deposition methods such as atomic layer deposition (ALD) have been suggested to fabricate the coating layer with controlled thickness and surface coverage (Gong et al., 2013; Shin et al., 2019; Zhang et al., 2020). However, the use of vacuum-based deposition for practical applications may be questionable because of cost-effectiveness and scalability issues.

In this study, we employed electrostatic spray deposition (ESD) to fabricate heterostructured electrodes because of its capability for the facile synthesis of nanostructures in a relatively simple process that is both cost-effective and scalable (Joshi et al., 2021). During the ESD process, suspension droplets are atomized to the sub-micron scale, allowing for the fabrication of a wide range of nanostructures with control of the spray parameters and suspension (Lee et al., 2019; Lee et al., 2021b; Joshi et al., 2021). We employed a coaxial nozzle for co-spraying the LSC powder and GDC precursor-based solution in the core and shell nozzles, respectively, to fabricate heterostructured LSC-GDC electrodes. Furthermore, the sol-gel combustion (SGC) reaction was induced in the ESD process by adding combustion fuel to the precursor solution to achieve a thin conformal coating layer with high crystallinity. LSC-GDC electrodes fabricated by SGC-assisted ESD exhibited greatly suppressed Sr segregation and therefore substantially improved long-term stability compared with the LSC electrode, exhibiting 63.5 times higher stability at 650°C for 300 h. In addition, the electrochemical performance was considerably improved owing to the facilitated surface exchange reactions with the heterointerfaces. Our results demonstrate a novel method to achieve highly durable SOFC cathodes by fabricating heterostructured electrodes using the modified ESD process.

## Experiment

### SGC-Assisted ESD

The La_0.6_Sr_0.4_CoO_3_ (LSC) nanopowder suspension was prepared in an EtOH solvent with 5 wt% commercial LSC nanopowder (K-ceracell, Korea), 3 wt% dispersant (Triton X-100, Merck, Germany), 1 wt% binder (polyvinyl butyral, PVB, Alfa Aesar, United States), and 1 wt% plasticizer (polyethylene glycol, PEG, Alfa Aesar, United States). The GDC precursor solution was prepared in a di-water and ethanol solution at a ratio of 6:4 with a molar concentration of 0.0025 M. To induce the SGC reaction, ethylene glycol was added to the solution with a metal nitrate/fuel molar ratio of 1.5 considering the oxidizing and reducing valences. A coaxial nozzle (NANO NC, Korea) was used to spray the LSC suspension at the core and GDC precursor solution at the shell. The ESD process was conducted at a distance of 4 cm while applying a voltage of 17 kV and heating the substrate to 250 °C. After the deposition, the LSC-GDC cathode was sintered at 900°C for 3 h.

### Symmetric and Single Cell Fabrication

The symmetric cell was fabricated on pelletized GDC (Rhodia, LSA) sintered at 1,500°C for 5 h. The ESD process was conducted on both sides of the GDC pellet, as previously described. The single cell was fabricated as an anode-supported cell. For anode support, NiO, GDC powders, and Poly (methyl methacrylate) (PMMA, 5 μm size) as a pore former were combined in a mass ratio of 6:4:0.8 and ball-milled for 3 days with dispersant Hypermer KD-6 (Croda Advanced materials). Then, the homogeneous anode powder was uniaxially pressed and sintered at 900°C for 3 h. Next, the anode functional layer with NiO and GDC powders in a 6:4 ratio and GDC powders were ball-milled for 3 days and spin-coated on the NiO-GDC anode pellet. The spin-coated NiO-GDC pellet was sintered at 1,400°C for 5 h to obtain the densified electrolyte.

### Characterization

The fabricated symmetric and single cells were tested in a custom-made test setup in the temperature range of 500–650°C with a spectrometer (GAMRY Reference 600, GAMRY Inc.). The symmetric cell was tested in ambient air, and the single cell was tested under a constant flow (200 sccm) of air and wet H_2_ (3% H_2_O) for the cathode and anode sides, respectively. The long-term stability was tested at a constant current density of 1 A/cm^2^ at 600°C for 100 h. The thermal behavior of the prepared solution was subjected to a thermogravimetric analysis (TGA, DC Q600). A structural analysis was conducted using field-emission scanning electron microscopy (FE-SEM, JSM7000F, JEOL) and transmission electron microscopy (TEM, JEM-ARM 200F, JEOL). Elemental mapping of LSC-GDC was conducted using energy dispersive X-ray spectroscopy in scanning TEM (STEM-EDS). The crystal structure was analyzed using X-ray diffraction (XRD, D8 ADVANCE, Bruker Corp.). X-ray photoelectron spectroscopy (XPS, ESCA Lab 250 XPS spectrometer, VG Scientific Instruments) was used for the analysis of segregated Sr.

## Results and Discussion

The use of a heterostructured electrode with a thin conformal coating of stable materials on the active electrode is a promising strategy to achieve high chemical and electrochemical stability of SOFC cathodes over long-term operation at elevated temperatures (Choi et al., 2018; Lee et al., 2021a). The coverage of the coating layer should be as extensive as possible to minimize the exposed electrode surface for high stability, while the coating layer should be sufficiently thin to minimize the detrimental effects of the less reactive coating layer for high performance (Choi et al., 2020a; Zhang et al., 2020). We employed EDS with coaxial nozzles to address these structural requirements because of its ability to produce well-controlled composite nanostructures. Moreover, the SGC process, which uses the oxidizer and fuel during the intense reduction and oxidation reactions that induce high thermal energy with a short reaction time, was employed to fabricate nanoparticles with high crystallinity and uniform size (Wattanasiriwech and Wattanasiriwech, 2013; Zarkov et al., 2016). The fabrication of a crystalline coating layer during deposition at a low temperature is particularly important for maintaining the heterostructured electrode after subsequent sintering at high temperatures because the free energy of the crystalline structure is lower than that of the amorphous structure. (Liu et al., 2014; Bretos et al., 2018). Two components are required to produce the metal oxide, namely metal nitrate as an oxidizer and fuel as a reducer, as described in reaction (1).

$$M{\left(NO_{3}\right)}_{x}\cdot nH_{2}O\left(\mathrm{oxidizer}\right)+\mathrm{Fuel}\left(\mathrm{reducer}\right)\to MO_{y}+N_{2}+H_{2}O+CO_{2}+\mathrm{Heat}$$ (1)

**FIGURE 1:**
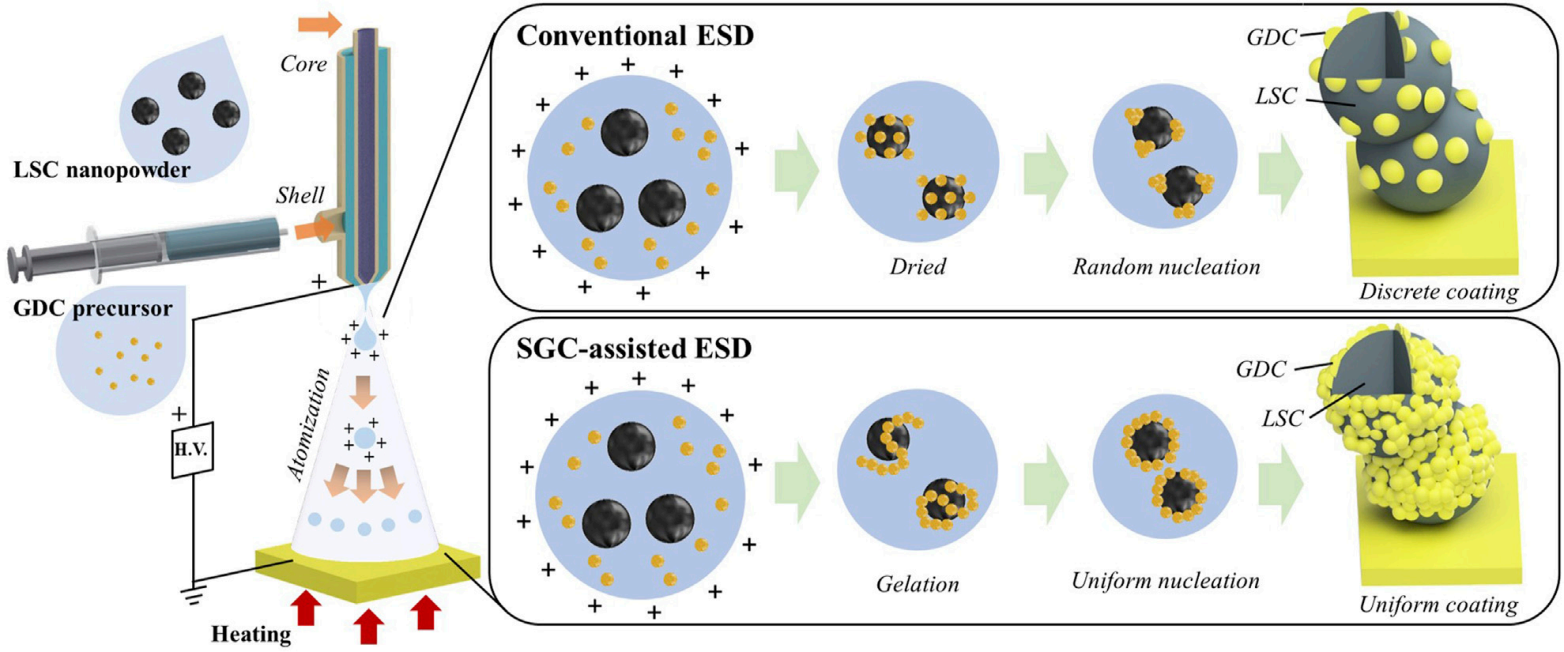
Process schematic of conventional ESD and sol-gel combustion-assisted ESD.

Figure 1 shows a schematic of the conventional ESD and SGC-assisted ESD processes. Two solutions with the LSC powders and GDC precursors were fed into the core and shell nozzles, respectively. The solution containing the GDC precursors contained Gd and Ce nitrate as oxidizers and ethylene glycol as a fuel for the SGC process. Typically, the SGC process is associated with gelation of the solution at lower temperatures ( ∼ 100°C) and combustion at higher temperatures (>150°C) (Wattanasiriwech and Wattanasiriwech, 2013; Zarkov et al., 2016). In this study, both processes can occur in a single step, because the droplet size in the ESD process is sufficiently small to be dried and gelled during the flight to the substrate, leading to continuous combustion reactions, while thermal energy is provided by heating the substrate. The gelation of the GDC precursor solution enables uniform nucleation on the LSC nanoparticle surface because of the linked structure with gelation; otherwise the GDC precursor solution nucleates at random locations to form clusters (Arachchige and Brock, 2007). The subsequent combustion reaction facilitates the crystallization of the coated GDC precursors without agglomeration owing to the short and intense thermal energy (Zarkov et al., 2016). Moreover, the combustion reactions in the sub-micron sized droplets can result in greater uniformity compared to the conventional ESD process because of the reduced reaction scale (Yu et al., 2015; Wang et al., 2016).

**FIGURE 2:**
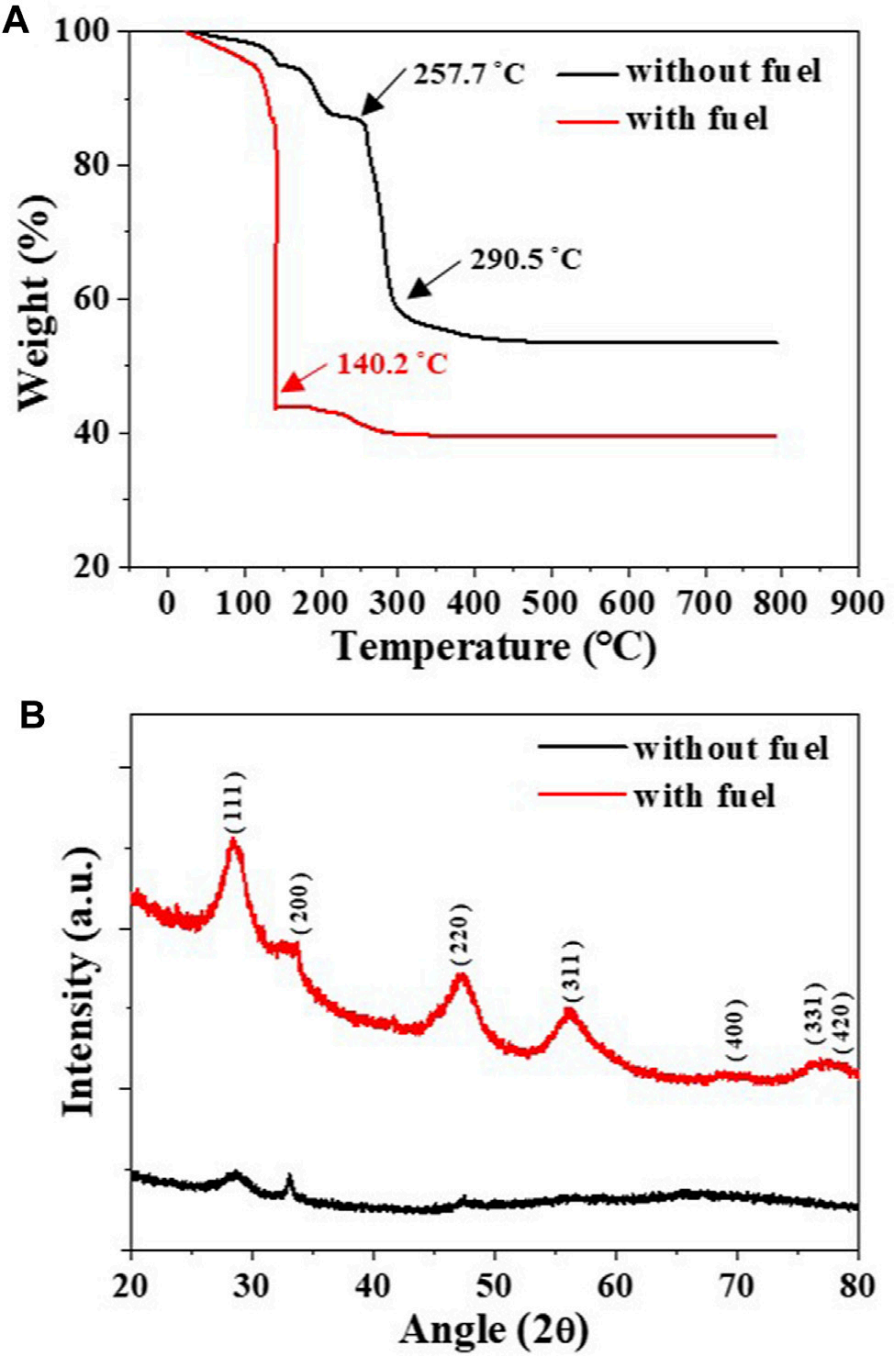
**(A)** TGA of the dried gels of GDC precursor solutions with and without fuel and **(B)** the XRD patterns of GDC powders after TGA measurement up to 600°C.

To verify the occurrence of the combustion reaction and its effects on crystallinity, two dried gels of GDC precursor solutions with and without the fuel were subjected to TGA, as shown in Figure 2A. The dried GDC gel without fuel shows a moderate weight loss at 257.7–290.5°C, indicating thermal decomposition of nitrate. However, the dried GDC gel with fuel shows a sharp weight loss at 140.2°C, indicating the presence of combustion reactions during the heat treatment (Wattanasiriwech and Wattanasiriwech, 2013; Zarkov et al., 2016). Figure 2B shows the XRD patterns of the GDC powders after TGA measurement up to 600°C. The GDC powder without fuel showed an amorphous-like structure owing to its temperature being lower than 400°C, the minimum typically required for crystallization (Prasad et al., 2008; Zarkov et al., 2016). Conversely, the GDC powders with fuel clearly showed a polycrystalline structure with an average grain size of 3.5 ± 5.2 nm. The crystallization of the GDC powder with fuel verifies that the prepared precursor solution with ethylene glycol is ready for SGC, providing additional thermal energy for the fabrication of nanocrystalline GDC powders.

**FIGURE 3:**
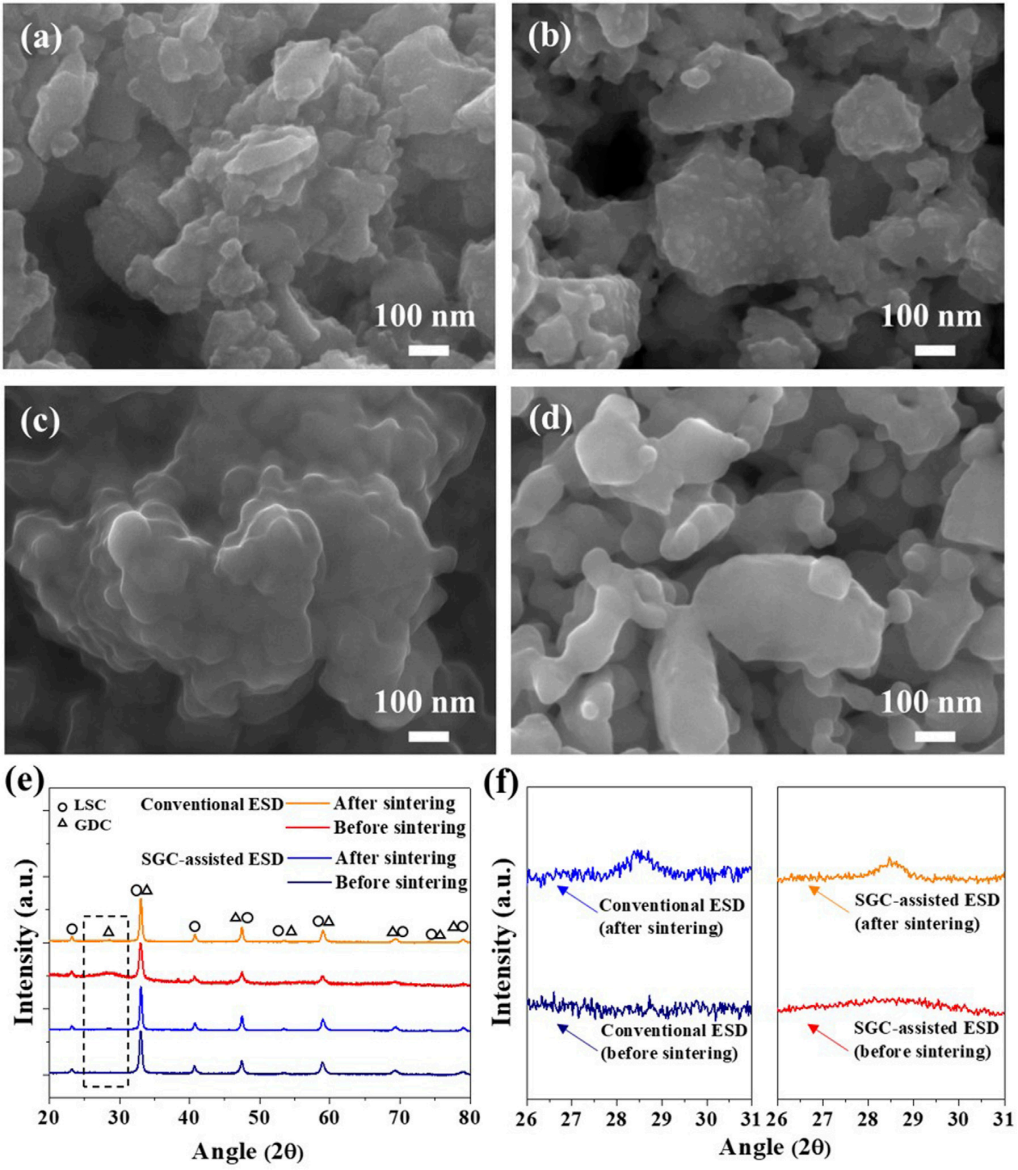
SEM images of LSC-GDC powders using conventional ESD **(A)** before and **(B)** after sintering at 800°C, and LSC-GDC powders using SGC-assisted ESD **(C)** before and **(D)** after sintering at 800°C. **(E,F)** XRD patterns of fabricated LSC-GDC powders.

**FIGURE 4:**
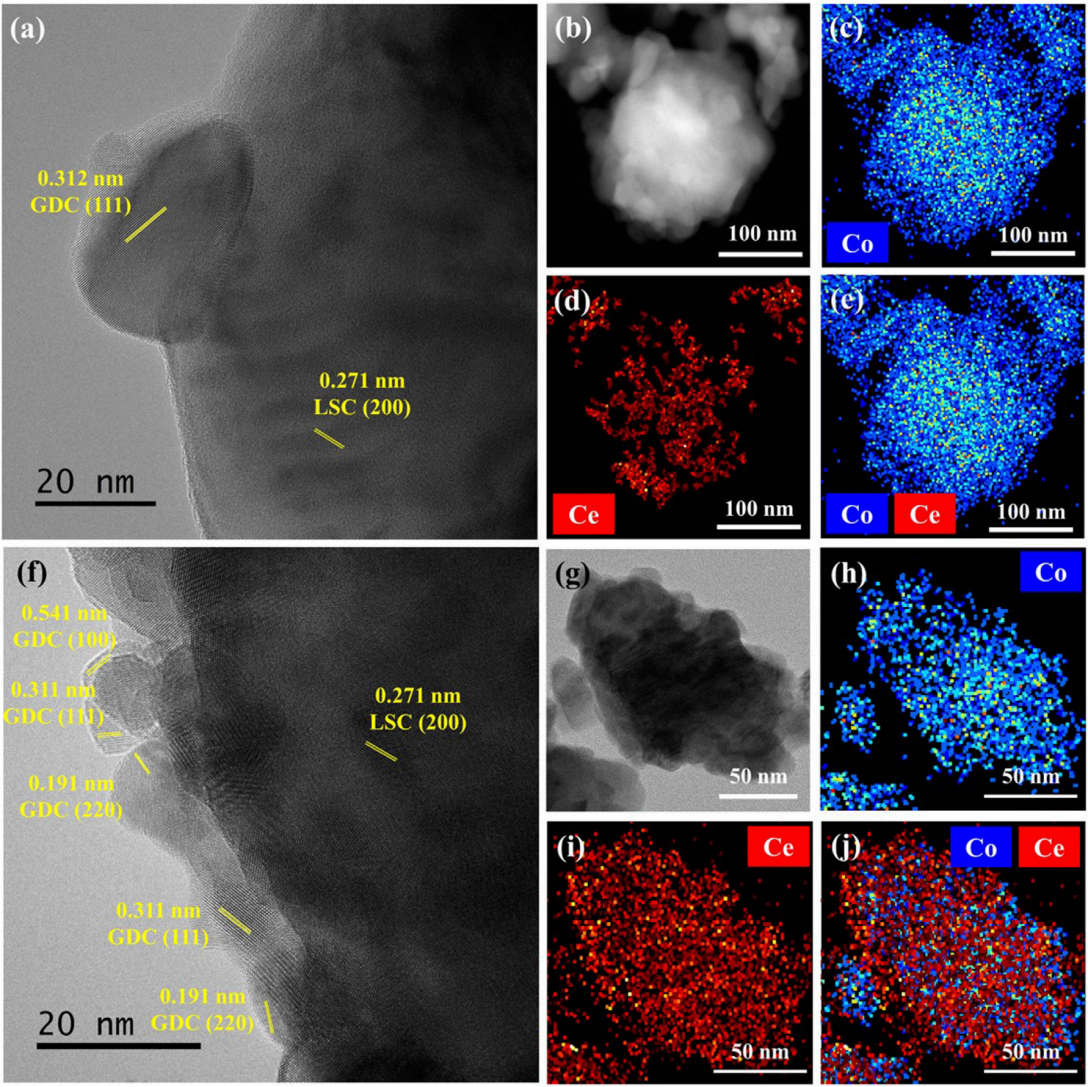
HR-TEM images of LSC-GDC powders prepared using **(A)** conventional ESD and **(F)** SGC-assisted ESD. TEM-Energy dispersive X-ray dispersive analysis (EDAX) elemental mapping of Co. and Ce for LSC-GDC powders using **(B–E)** conventional ESD and **(G–J)** SGC-assisted ESD.

Next, a coaxial nozzle was employed to fabricate the LSC-GDC powder using the core nozzle for the LSC powders and the shell nozzle for the GDC precursors with the fuel, while heating the substrate at 250°C to induce the SGC process. Figures 3A–D show SEM images of the LSC-GDC powders using conventional and SGC-assisted ESD before and after sintering at 800°C. The LSC-GDC powders fabricated by the conventional ESD show randomly dispersed GDC phases on the LSC surface with a size of 27.8 ± 5 nm and a surface coverage of 27.5% after sintering at 800°C. In contrast, the LSC-GDC powders fabricated by SGC-assisted ESD showed smooth surfaces before and after sintering at 800°C. Figures 3E,F show XRD patterns of LSC-GDC powders in a range from 20° to 80° for all GDC and LSC peaks and in a range from 26° to 31° for the GDC 111) peak. In the LSC-GDC powders fabricated by conventional ESD, the GDC 111) peak appeared after sintering at 800°C, whereas it was not noticeable before sintering. However, in the LSC-GDC powders fabricated by SGC-assisted ESD, the GDC 111) peak appeared before sintering and became sharper after sintering at 800°C. Therefore, heterostructured LSC-GDC powders with distinct structural features can be fabricated in discrete nanoparticles using conventional ESD without fuel, and conformal crystalline nanoparticles can be fabricated using SGC-assisted ESD with fuel in the precursor solution. Figure 4 shows HR-TEM images and elemental mappings to verify the distribution of the GDC phases in a high resolution. In the elemental mapping, the LSC and GDC phases are represented by Co. and Ce, respectively. Consistent with the SEM images, the LSC-GDC powders fabricated by conventional ESD show GDC nanoparticles with a size of 20–30 nm, which are discretely distributed on the LSC surface. In contrast, the LSC-GDC powders fabricated by the SGC-assisted ESD process showed conformally distributed GDC nanoparticles with a size of 8–10 nm in a thickness of 6.4 ± 2.8 nm. These results confirm that the SGC-assisted ESD process can successfully fabricate heterostructured nanoparticles with sufficiently high surface coverage. During the flight to the substrate, the GDC precursors in the droplet with the fuel were gelled to conformally cover the LSC nanoparticles and form crystalline GDC nanoparticles with high surface coverage upon the short intense combustion reaction. In contrast, the GDC precursors in the droplet without fuel agglomerated before crystallization to form discrete nanoparticles with low surface coverage. Moreover, the lower reaction scale in the ESD process compared with that in the conventional sol-gel process is another important factor that enables the conformal coating. Supplementary Figure S1 shows the irregular structures of the LSC-GDC powders fabricated by the bulk sol-gel process using a solution identical to that used for SGC-assisted ESD. In addition, the thickness of GDC can be precisely controlled by adjusting the flow rates of the two solutions in the SGC-assisted ESD to avoid performance degradation due to the lower surface activity of GDC relative to LSC. The total flow rate was fixed to 20 μl/min in this study and the flow rate ratios of the LSC powder to the GDC precursor solutions were controlled to be 20:0, 17.5:2.5, 15:5, 12.5:7.5, and 10:10, corresponding GDC loading of 0, 0.39, 0.92, 1.65, and 2.76 wt%, respectively. Supplementary Figure S2 shows SEM images of the LSC-GDC powders fabricated by controlling the GDC content in the solution using conventional and SGC-assisted ESD. For LSC-GDC powders fabricated by conventional ESD, the surface coverage of GDC nanoparticles increased with the GDC content, blocking the reaction sites for surface oxygen exchange reactions owing to the large particle sizes. However, for LSC-GDC powders fabricated by SGC-assisted ESD, the GDC nanoparticles fully covered the LSC particles regardless of the GDC content, while the thickness varied with the GDC content. Supplementary Figure S3 shows that the thickness of the GDC particles increased to 13 ± 2.8 nm with a GDC loading of 2.76 wt% as compared with the thickness of 6.4 ± 2.1 nm with a GDC loading of 0.92 wt%, substantiating that the thickness can be controlled by the GDC content in the solution.

**FIGURE 5:**
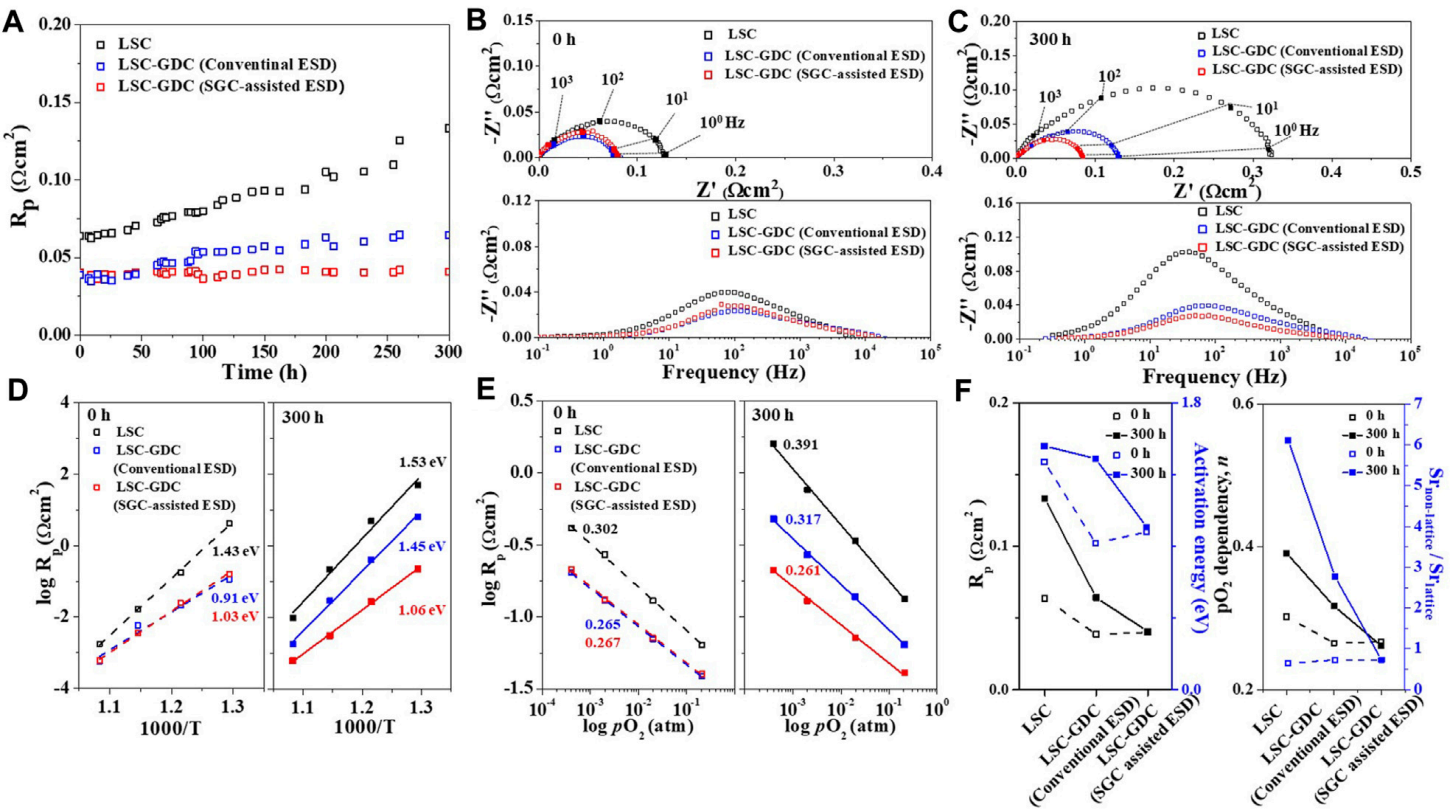
Electrochemical evaluation of the LSC electrode and LSC-GDC electrodes fabricated by the conventional ESD and SGC-assisted ESD. **(A)** Polarization resistance with respect to time for 300 h at 650°C. Nyquist and Bode plots at **(B)** 0 h and **(C)** 300 h. **(D)** Arrhenius plots at 0 and 300 h. **(E)** Polarization resistance as a function of oxygen partial pressure at 650°C. **(F)** Polarization resistance, activation energy, *p*O_2_ dependency *n*, and Sr_non-lattice_/Sr_lattice_ ratio for the three electrodes.

The electrochemical stability of the heterostructured LSC-GDC electrodes was evaluated in the temperature range of 500–650°C in a symmetric cell configuration to focus exclusively on the electrode. The GDC content was optimized for the LSC-GDC electrodes to maximize their performance (Supplementary Figure S4). Figure 5A compares the polarization resistance (R_p_) values of the LSC electrode and LSC-GDC electrodes fabricated by conventional ESD and SSG-assisted ESD at 650°C for 300 h. The LSC electrode shows a significant degradation, resulting in R_p_ more than doubling after 300 h. In contrast, both LSC-GDC electrodes exhibit substantially improved electrochemical stability. In particular, the R_p_ value of the LSC-GDC electrode fabricated by SSG-assisted ESD remained almost unchanged after 300 h, verifying the effects of the heterostructures with thin conformal coatings in enhancing electrochemical stability. The remarkably improved electrochemical stability of the LSC-GDC electrodes can be attributed to the conformal coating of the relatively stable material, GDC, preventing the degradation of the electrode reactivity toward ORR kinetics. Figures 5B,C show the Nyquist plots and corresponding Bode plots of the LSC and LSC-GDC electrodes before and after the stability test at 650°C for 300 h. The prominent increase in the imaginary component in the frequency range 10^1^–10^2^ Hz concurrent with the increase in the R_p_ value of the LSC electrode confirms that the electrochemical degradation mainly originates from the deteriorated ORR kinetics at the electrode surface. At the same time, the negligible increase in the imaginary component in the same frequency range and the unchanged R_p_ value of the LSC-GDC electrode fabricated by SSG-assisted ESD confirm that the thin conformal GDC coating layer can effectively prevent the degradation of the electrode surface. Interestingly, the stability of the LSC-GDC electrode fabricated by conventional ESD was also improved, but by a smaller margin than that of the LSC-GDC electrode fabricated by SSG-assisted ESD. The increase in the imaginary component in the same frequency range as the LSC electrode indicates that the degradation originates from the exposed electrode surface owing to the lower surface coverage of the GDC coating layer.

The electrode performance was also improved in the LSC-GDC electrodes. Figures 5B,C show that the R_p_ values of LSC-GDC electrodes were reduced by 37–38% for the LSC electrode, from 0.064 Ωcm^2^ to 0.039–0.040 Ωcm^2^; this and the decrease in the imaginary component in the frequency range of 10^1^–10^2^ Hz illustrate the improved ORR kinetics at the surface. Figure 5D shows the considerable reduction in the activation energy for the polarization resistance, from 1.43 eV for the LSC electrode to 0.91 and 1.03 eV for the LSC-GDC electrodes fabricated by conventional ESD and SSG-assisted ESD, respectively, verifying that the improved electrode performance can be attributed not only to the extended reaction sites but also to the change in the rate-determining step (RDS). Given the typical E_a_ values of 1.4–1.5 eV for the surface exchange reactions and 0.9–1.0 eV for oxygen ion transport into the electrolyte, the improved electrode performance can be attributed to the facilitated surface exchange reactions with extended reaction sites due to the LSC-GDC heterointerfaces (Baumann et al., 2006; Donazzi et al., 2015; Lee et al., 2019). Figure 5E shows the dependency of the R_p_ values on the oxygen partial pressure according to Equation 2, which further supports the RDS changes in the LSC-GDC electrodes.

$$R_{p}\propto pO_{2}^{-n}$$ (2)

The value of *n* decreased from 0.302 for the LSC electrode to 0.265–0.267 for the LSC-GDC electrodes, indicating that the RDS changed from the charge transfer at the cathode surface (*n* ∼ 3/8) to the charge transfer at the triple-phase boundaries (*n* ∼ 1/4), consistent with the results from the Bode plot (Figure 5B) and Arrhenius plot (Figure 5D) (Lee et al., 2019). These results confirm that the GDC coating layer on the LSC electrode can facilitate surface exchange reactions with extended reaction sites. However, GDC content higher than the optimum loading leads to higher R_p_ and E_a_ values due to the loss of reaction sites by blocking the open pores and reducing the reactive area (Supplementary Figure S4).

More importantly, a comparison of the E_a_ and *n* values before and after the stability test at 650°C for 300 h (Figures 5D,E) provides clear evidence that the thin conformal GDC coating layer can effectively prevent electrode degradation. The significant increase in the R_p_ value of the LSC electrode, accompanied by an increase in the E_a_ and *n* values, confirms that the degradation is mainly induced by the deteriorated surface activity. In contrast, insignificant changes in the R_p_, E_a_, and *n* values of the LSC-GDC electrode fabricated by SSG-assisted ESD confirm that the surface activity is well preserved by the GDC coating layer. Furthermore, the LSC-GDC electrode fabricated by conventional ESD showed similar R_p_, E_a_, and *n* values to the LSC-GDC electrode fabricated by SSG-assisted ESD before the stability test, but all values increased after the test, approaching those of the LSC electrode. This indicates that a similar degradation to the LSC electrode occurs in the LSC-GDC electrode fabricated by conventional ESD because of the insufficient surface coverage, demonstrating the importance of the SGC process to enable a thin conformal coating with substantially high surface coverage.

**FIGURE 6:**
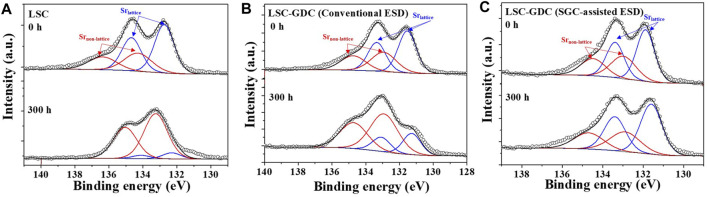
XPS spectra of Sr 3d peak before and after the stability test for 300 h **(A)** LSC electrode, **(B)** LSC-GDC electrode fabricated by conventional ESD, and **(C)** LSC-GDC electrode fabricated by SGC-assisted ESD.

Cation segregation toward the surface of perovskite oxides induces substantial changes in the surface chemistry and structure, resulting in significant degradation of electrochemical performance (Choi et al., 2021; Koo et al., 2018; Myung et al., 2016; Neagu et al., 2013). In particular, Sr segregation leads to non-stoichiometry and the formation of secondary phases at the surface, which inhibit charge transfer and surface exchange reactions (Lee et al., 2013). To verify the changes in the surface chemistry, A XPS analysis was conducted before and after the stability test, as shown in Figures 6A–C. The Sr 3d peaks were deconvoluted into two peaks corresponding to Sr in the lattice (Sr_lattice_) and Sr in the non-lattice (Sr_non-lattice_) (Chen et al., 2018; Choi et al., 2020a). Initially, the three electrodes showed similar Sr_non-lattice_/Sr_lattice_ ratios of 0.645–0.726. However, after the stability test at 650°C for 300 h, the Sr_non-lattice_/Sr_lattice_ ratios increased significantly to 6.11 and 2.76 for the LSC electrode and LSC-GDC fabricated by conventional ESD, respectively, indicating that Sr segregation beyond the solubility limit of the perovskite oxide induced the formation of secondary phases, such as SrO_x_ and Sr(OH)_x_ (Cai et al., 2012; Chen et al., 2018; Choi et al., 2020a). In contrast, the Sr_non-lattice_/Sr_lattice_ ratio remained unchanged at 0.720 in the LSC-GDC electrode fabricated by SGC-assisted ESD, confirming that Sr segregation is effectively prevented.

Figure 5F compares the results of the electrochemical analyses (R_p_, E_a_ for R_p_, and *n* values) and chemical analyses (Sr_non-lattice_/Sr_lattice_ ratio) on the three electrodes. The LSC electrode showed a substantially increased R_p_ value after the stability test, which was accompanied by an increase in activation energy for R_p_ and pO_2_ dependency, *n*, and Sr_non-lattice_/Sr_lattice_ ratio, verifying that the surface oxygen exchange reactivity has deteriorated by Sr segregation toward the electrode surface (Choi et al., 2020a). In contrast, the LSC-GDC electrode fabricated by SGC-assisted ESD maintained its initial chemical and electrochemical properties. Moreover, despite this clear improvement, the LSC-GDC electrode fabricated by conventional ESD showed considerable degradation, and the increase in the Sr_non-lattice_/Sr_lattice_ ratio after the stability test indicates that this originates from the exposed electrode surface owing to insufficient surface coverage. Therefore, the chemical and electrochemical analyses so far clearly indicate that the heterostructured electrode can greatly suppress degradation induced by Sr segregation, and furthermore, that SGC-assisted ESD can provide an effective fabrication method to enable a thin conformal coating on the electrodes.

**FIGURE 7:**
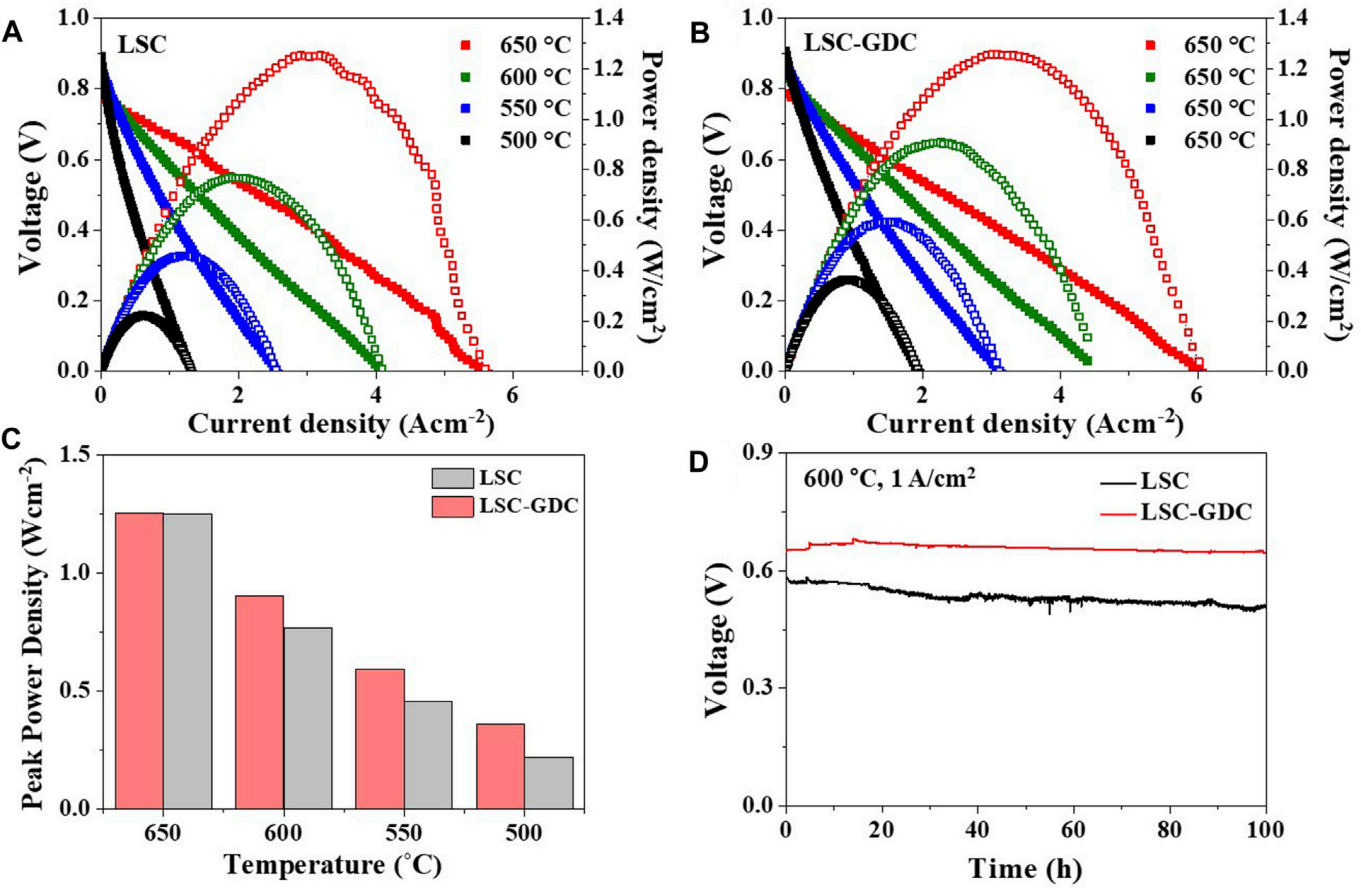
Electrochemical performance of single cells with **(A)** LSC electrode and **(B)** LSC-GDC electrode fabricated by SGC-assisted ESD. **(C)** Comparison of the peak power densities in a temperature range from 500 to 650°C. **(D)** Stability test with a constant current density of 1 A/cm^2^ at 600°C for 100 h.

The electrochemical performance and stability of the LSC electrode and the LSC-GDC electrode fabricated by SGC-assisted ESD were evaluated in a Ni-GDC anode-supported single cell configuration. A cross-sectional image of the single cell is shown in Supplementary Figure S5. Figures 7A,B show the I–V curves of the 2 cells in the temperature range of 650–500°C. At all temperatures tested in this study, the LSC-GDC electrode fabricated by SGC-assisted ESD exhibited a higher peak power density than the LSC electrode. Moreover, the performance improvement of the LSC-GDC electrode fabricated by SGC-assisted ESD was more pronounced at lower temperatures. Figure 7C shows that the peak power density of the LSC-GDC electrode fabricated by SGC-assisted ESD was greater by 1.6% at 650°C (1.21 W/cm^2^) than that of the LSC electrode, and greater by 63.2% at 500°C (0.36 W/cm^2^). Supplementary Figure S6 shows that R_p_ values of the LSC-GDC electrode fabricated by SGC-assisted ESD were also reduced further at lower temperatures; for example, the R_p_ value of the LSC-GDC electrode fabricated by SGC-assisted ESD was smaller by 22.3% at 650°C (0.0053 Ωcm^2^) than that of LSC electrode, and smaller by 47.8% at 500°C (0.96 Ωcm^2^). The more pronounced improvement in the electrochemical performance at lower temperatures can be attributed to the lower E_a_ for R_p_ of the LSC-GDC electrode fabricated by SGC-assisted ESD (1.21 eV) compared with that of the LSC electrode (1.35 eV). The E_a_ values in the single cell configuration did not exactly match those in the symmetric cell configuration because of the contribution of the anode polarization resistance. However, the clear temperature dependency, that is, the more pronounced improvement at lower temperatures, verifies the facilitated ORR kinetics at the interfaces in the heterostructured LSC-GDC electrode. Figure 7D shows galvanostatic measurements with a constant current density of 1 A/cm^2^ at 600°C for 100 h to compare the electrochemical stability. The initial voltage was maintained almost unchanged in the LSC-GDC electrode fabricated by SGC-assisted ESD ( ∼ 0.3% reduction in the voltage), whereas it was reduced by 12.3% in the LSC electrode. These results demonstrate the excellent thermal stability of the LSC-GDC electrode fabricated by SGC-assisted ESD, confirming that high performance and stability in IT-SOFCs can be achieved with the thin conformal coating of the GDC layer using a wet chemical-based SGC-assisted ESD process.

## Conclusion

We demonstrated a sol-gel combustion-assisted ESD process with a coaxial nozzle to fabricate heterostructured electrodes for durable SOFC operation. The GDC precursor solution was sol-gel combusted during the ESD process to form a thin conformal coating layer on the LSC nanoparticles. Degradation induced by Sr segregation was greatly suppressed in the LSC-GDC electrode fabricated by SGC-assisted ESD, exhibiting excellent stability in both symmetric and single cell configurations. A comparison with the LSC-GDC electrode fabricated by conventional ESD verified that the high surface coverage, that is, the thin conformal coating, is critical to achieve high stability in heterostructured electrodes. Moreover, the polarization resistance decreased in the LSC-GDC electrode fabricated by SGC-assisted ESD due to the facilitated ORR kinetics at the interfaces in the heterostructured electrode. Our results provide a novel method for fabricating highly stable SOFC electrodes with nanostructured electrodes in a single step.

## Data Availability

The original contributions presented in the study are included in the article/Supplementary Material, further inquiries can be directed to the corresponding author.

## References

[B1] Arachchige I. U. Brock S. L. (2007). Sol-Gel Methods for the Assembly of Metal Chalcogenide Quantum Dots. Acc. Chem. Res. 40, 801–809. 10.1021/ar600028s 17441681

[B2] Baumann F. Fleig J. Habermeier H. Maier J. (2006). Impedance Spectroscopic Study on Well-Defined (La,Sr)(Co,Fe)O3−δ Model Electrodes. Solid State Ionics 177, 1071–1081. 10.1016/j.ssi.2006.02.045

[B3] Bretos I. Jiménez R. Ricote J. Calzada M. L. (2018). Low-temperature Crystallization of Solution-Derived Metal Oxide Thin Films Assisted by Chemical Processes. Chem. Soc. Rev. 47, 291–308. 10.1039/c6cs00917d 29165444

[B4] Cai Z. Kubicek M. Fleig J. Yildiz B. (2012). Chemical Heterogeneities on La0.6Sr0.4CoO3−δ Thin Films-Correlations to Cathode Surface Activity and Stability. Chem. Mater. 24, 1116–1127. 10.1021/cm203501u

[B5] Chen Y. Jung W. Cai Z. Kim J. J. Tuller H. L. Yildiz B. (2012). Impact of Sr Segregation on the Electronic Structure and Oxygen Reduction Activity of SrTi1−xFexO3 Surfaces. Energ. Environ. Sci. 5, 7979–7988. 10.1039/c2ee21463f

[B6] Chen Y. Chen Y. Ding D. Ding Y. Choi Y. Zhang L. (2017). A Robust and Active Hybrid Catalyst for Facile Oxygen Reduction in Solid Oxide Fuel Cells. Energy Environ. Sci. 10, 964–971. 10.1039/c6ee03656b

[B7] Chen H. Guo Z. Zhang L. A. Li Y. Li F. Zhang Y. (2018). Improving the Electrocatalytic Activity and Durability of the La0.6Sr0.4Co0.2Fe0.8O3−δ Cathode by Surface Modification. ACS Appl. Mater. Inter. 10, 39785–39793. 10.1021/acsami.8b14693 30372019

[B8] Choi M. Lee J. Lee W. (2018). Nano-film Coated Cathode Functional Layers towards High Performance Solid Oxide Fuel Cells. J. Mater. Chem. A. 6, 11811–11818. 10.1039/c8ta01660g

[B9] Choi M. Ibrahim I. A. M. Kim K. Koo J. Y. Kim S. J. Son J.-W. (2020a). Engineering of Charged Defects at Perovskite Oxide Surfaces for Exceptionally Stable Solid Oxide Fuel Cell Electrodes. ACS Appl. Mater. Inter. 12, 21494–21504. 10.1021/acsami.9b21919 32315147

[B10] Choi M. Kim S. Paik J. Lee W. (2020b). Enhanced Cr Tolerance of Perovskite Oxide via Gd0.1Ce0.9O2 Surface Modifications. Korean J. Chem. Eng. 37, 1346–1351. 10.1007/s11814-020-0562-x

[B11] Choi M. Kim S. J. Lee W. (2021). Effects of Water Atmosphere on Chemical Degradation of PrBa0.5Sr0.5Co1.5Fe0.5O5+δ Electrodes. Ceramics Int. 47, 7790–7797. 10.1016/j.ceramint.2020.11.124

[B12] Choi M. Lee W. (2021). Tuning the Oxygen Vacancy Concentration in a Heterostructured Electrode for High Chemical and Electrochemical Stabilities. Chem. Eng. J. 431, 134345. 10.1016/j.cej.2021.134345

[B13] Ding D. Li X. Lai S. Y. Gerdes K. Liu M. (2014). Enhancing SOFC Cathode Performance by Surface Modification through Infiltration. Energ. Environ. Sci. 7, 552–575. 10.1039/c3ee42926a

[B14] Donazzi A. Pelosato R. Cordaro G. Stucchi D. Cristiani C. Dotelli G. (2015). Evaluation of Ba Deficient NdBaCo2O5+δ Oxide as Cathode Material for IT-SOFC. Electrochimica Acta 182, 573–587. 10.1016/j.electacta.2015.09.117

[B15] Gong Y. Palacio D. Song X. Patel R. L. Liang X. Zhao X. (2013). Stabilizing Nanostructured Solid Oxide Fuel Cell Cathode with Atomic Layer Deposition. Nano Lett. 13, 4340–4345. 10.1021/nl402138w 23924170

[B16] Hamada I. Uozumi A. Morikawa Y. Yanase A. Katayama-Yoshida H. (2011). A Density Functional Theory Study of Self-Regenerating Catalysts LaFe1-xMxO3-Y (M = Pd, Rh, Pt). J. Am. Chem. Soc. 133, 18506–18509. 10.1021/ja110302t 22026920

[B17] Jalili H. Han J. W. Kuru Y. Cai Z. Yildiz B. (2011). New Insights into the Strain Coupling to Surface Chemistry, Electronic Structure, and Reactivity of La0.7Sr0.3MnO3. J. Phys. Chem. Lett. 2, 801–807. 10.1021/jz200160b

[B18] Joshi B. Samuel E. Kim Y. I. Yarin A. L. Swihart M. T. Yoon S. S. (2021). Electrostatically Sprayed Nanostructured Electrodes for Energy Conversion and Storage Devices. Adv. Funct. Mater. 31, 2008181. 10.1002/adfm.202008181

[B19] Kawada T. Suzuki J. Sase M. Kaimai A. Yashiro K. Nigara Y. (2002). Determination of Oxygen Vacancy Concentration in a Thin Film of La[sub 0.6]Sr[sub 0.4]CoO[sub 3−δ] by an Electrochemical Method. J. Electrochem. Soc. 149, E252. 10.1149/1.1479728

[B20] Koo J. Y. Kwon H. Ahn M. Choi M. Son J.-W. Han J. W. (2018). Suppression of Cation Segregation in (La,Sr)CoO3−δ by Elastic Energy Minimization. ACS Appl. Mater. Inter. 10, 8057–8065. 10.1021/acsami.7b19390 29443491

[B21] Lee H. B. Prinz F. B. Cai W. (2010). Atomistic Simulations of Surface Segregation of Defects in Solid Oxide Electrolytes. Acta Materialia 58, 2197–2206. 10.1016/j.actamat.2009.12.005

[B22] Lee W. Han J. W. Chen Y. Cai Z. Yildiz B. (2013). Cation Size Mismatch and Charge Interactions Drive Dopant Segregation at the Surfaces of Manganite Perovskites. J. Am. Chem. Soc. 135, 7909–7925. 10.1021/ja3125349 23642000

[B23] Lee D. Lee Y.-L. Grimaud A. Hong W. T. Biegalski M. D. Morgan D. (2014). Enhanced Oxygen Surface Exchange Kinetics and Stability on Epitaxial La0.8Sr0.2CoO3−δ Thin Films by La0.8Sr0.2MnO3−δ Decoration. J. Phys. Chem. C 118, 14326–14334. 10.1021/jp502192m

[B24] Lee J. Hwang S. Ahn M. Choi M. Han S. Byun D. (2019). Enhanced Interface Reactivity by a Nanowrinkled Functional Layer for Intermediate-Temperature Solid Oxide Fuel Cells. J. Mater. Chem. A. 7, 21120–21127. 10.1039/c9ta04818a

[B25] Lee J. Choi M. Lee W. (2021a). Encapsulation of Metal Catalysts for Stable Solid Oxide Fuel Cell Cathodes. Int. J. Precision Eng. Manufacturing-Green Tech. 8, 1–7. 10.1007/s40684-020-00290-8

[B26] Lee J. Koo H. Kim S. Y. Kim S. J. Lee W. (2021b). Electrostatic spray Deposition of Chemochromic WO3-Pd Sensor for Hydrogen Leakage Detection at Room Temperature. Sensors Actuators B: Chem. 327, 128930. 10.1016/j.snb.2020.128930

[B27] Liu J. Xu X. Brush L. Anantram M. P. (2014). A Multi-Scale Analysis of the Crystallization of Amorphous Germanium Telluride Using Ab *Initio* Simulations and Classical Crystallization Theory. J. Appl. Phys. 115, 023513. 10.1063/1.4861721

[B28] Lynch M. E. Yang L. Qin W. Choi J.-J. Liu M. Blinn K. (2011). Enhancement of La0.6Sr0.4Co0.2Fe0.8O3-δ Durability and Surface Electrocatalytic Activity by La0.85Sr0.15MnO3±δ Investigated Using a New Test Electrode Platform. Energ. Environ. Sci. 4, 2249–2258. 10.1039/c1ee01188j

[B29] Myung J.-H. Neagu D. Miller D. N. Irvine J. T. S. (2016). Switching on Electrocatalytic Activity in Solid Oxide Cells. Nature 537, 528–531. 10.1038/nature19090 27548878

[B30] Neagu D. Tsekouras G. Miller D. N. Ménard H. Irvine J. T. S. (2013). *In Situ* Growth of Nanoparticles through Control of Non-stoichiometry. Nat. Chem 5, 916–923. 10.1038/nchem.1773 24153368

[B31] Prasad D. H. Son J.-W. Kim B.-K. Lee H.-W. Lee J.-H. (2008). Synthesis of Nano-Crystalline Ce0.9Gd0.1O1.95 Electrolyte by Novel Sol-Gel Thermolysis Process for IT-SOFCs. J. Eur. Ceram. Soc. 28, 3107–3112. 10.1016/j.jeurceramsoc.2008.05.021

[B32] Shin J. W. Oh S. Lee S. Yu J.-G. Park J. Go D. (2019). Ultrathin Atomic Layer-Deposited CeO2 Overlayer for High-Performance Fuel Cell Electrodes. ACS Appl. Mater. Inter. 11, 46651–46657. 10.1021/acsami.9b10572 31697463

[B33] Wang B. Yu X. Guo P. Huang W. Zeng L. Zhou N. (2016). Solution-Processed All-Oxide Transparent High-Performance Transistors Fabricated by Spray-Combustion Synthesis. Adv. Electron. Mater. 2, 1500427. 10.1002/aelm.201500427

[B34] Wattanasiriwech D. Wattanasiriwech S. (2013). Effects of Fuel Contents and Surface Modification on the Sol-Gel Combustion Ce0.9 Gd0.1O1.95 Nanopowder. Energ. Proced. 34, 524–533. 10.1016/j.egypro.2013.06.781

[B35] Wen Y. Yang T. Lee D. Lee H. N. Crumlin E. J. Huang K. (2018). Temporal and thermal Evolutions of Surface Sr-Segregation in Pristine and Atomic Layer Deposition Modified La0.6Sr0.4CoO3−δ Epitaxial Films. J. Mater. Chem. A. 6, 24378–24388. 10.1039/c8ta08355j

[B36] Yu X. Smith J. Zhou N. Zeng L. Guo P. Xia Y. (2015). Spray-combustion Synthesis: Efficient Solution Route to High-Performance Oxide Transistors. Proc. Natl. Acad. Sci. U.S.A. 112, 3217–3222. 10.1073/pnas.1501548112 25733848 PMC4371916

[B37] Zarkov A. Stanulis A. Salkus T. Kezionis A. Jasulaitiene V. Ramanauskas R. (2016). Synthesis of Nanocrystalline Gadolinium Doped Ceria via Sol-Gel Combustion and Sol-Gel Synthesis Routes. Ceramics Int. 42, 3972–3988. 10.1016/j.ceramint.2015.11.066

[B38] Zhang Y. Wen Y. Huang K. Nicholas J. D. (2020). Atomic Layer Deposited Zirconia Overcoats as On-Board Strontium Getters for Improved Solid Oxide Fuel Cell Nanocomposite Cathode Durability. ACS Appl. Energ. Mater. 3, 4057–4067. 10.1021/acsaem.0c00558

