# Supplementary material for: Sol-Gel Combustion-Assisted Electrostatic Spray Deposition for Durable Solid Oxide Fuel Cell Cathodes
Source: Front Chem. 2022 Apr 11;10:873758. doi: 10.3389/fchem.2022.873758 (PMC9035593; doi:10.3389/fchem.2022.873758)
Supplement: Supplementary file 1 [file DataSheet1.docx]

Supporting Materials

Sol-gel combustion-assisted electrostatic spray deposition for durable solid oxide fuel cell cathodes

Jongseo Lee^1^, Sehee Bang^2^, Wonyoung Lee^2^*

^1^Advanced Defense Science & Technology Research Institute, Agency for Defense Development, Daejeon 34186, Republic of Korea

^2^School of Mechanical Engineering, Sungkyunkwan University, Suwon 16419, Republic of Korea

*Corresponding author: leewy@skku.edu.


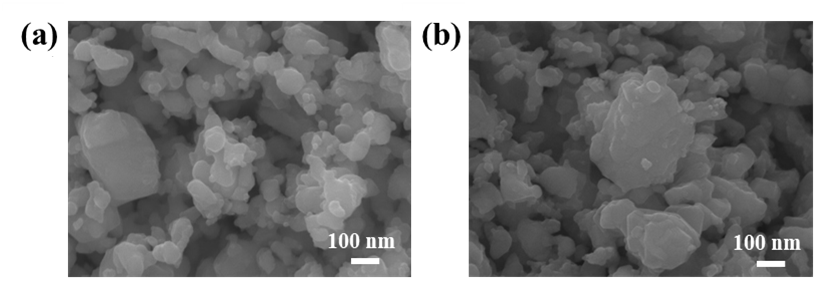


**Supplementary Figure 1.** Sol-gel combusted LSC-GDC powder. (a) As fabricated and (b) sintered at 800 °C


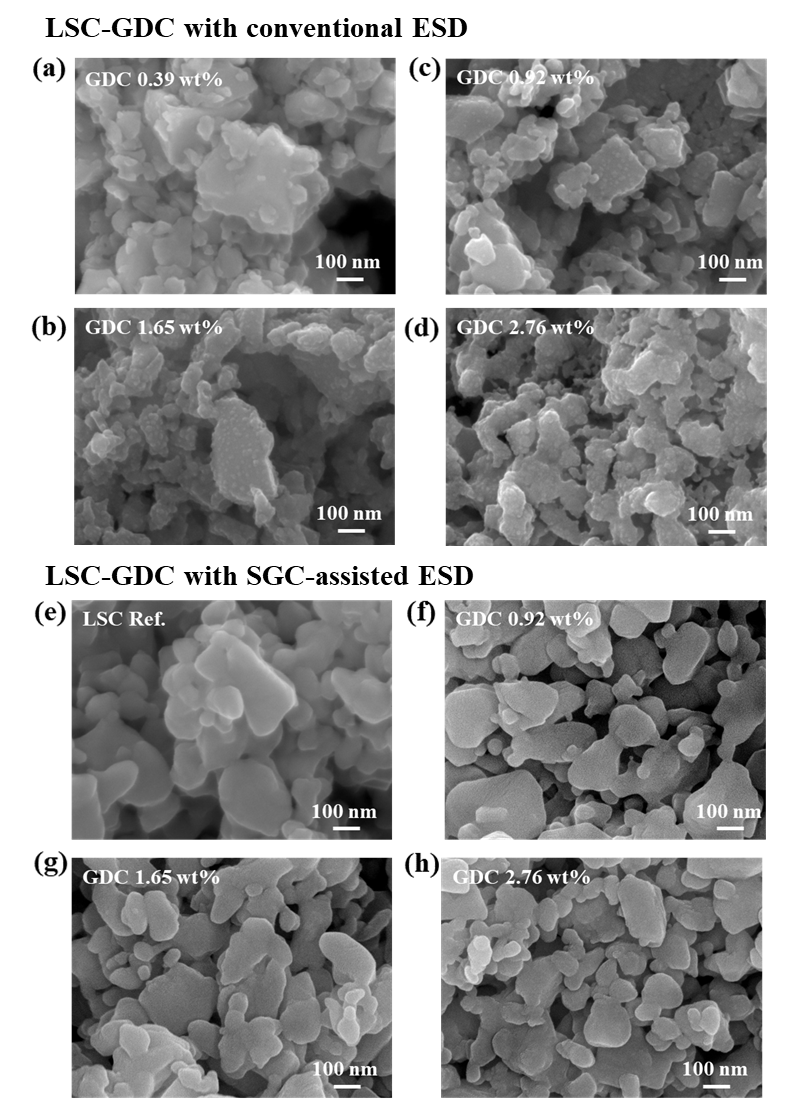


**Supplementary Figure 2.** SEM images of LSC-GDC with (a–d) conventional ESD and (e–h) SGC-assisted ESD for GDC content of 0.39, 0.92, 1.65, and 2.76 wt%


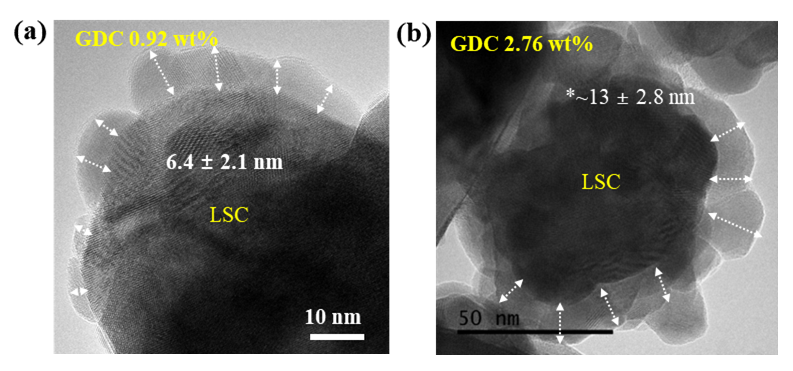


**Supplementary Figure 3.** TEM images of LSC-GDC with SGC-assisted ESD for GDC content of (a) 0.92 and (b) 2.76 wt%


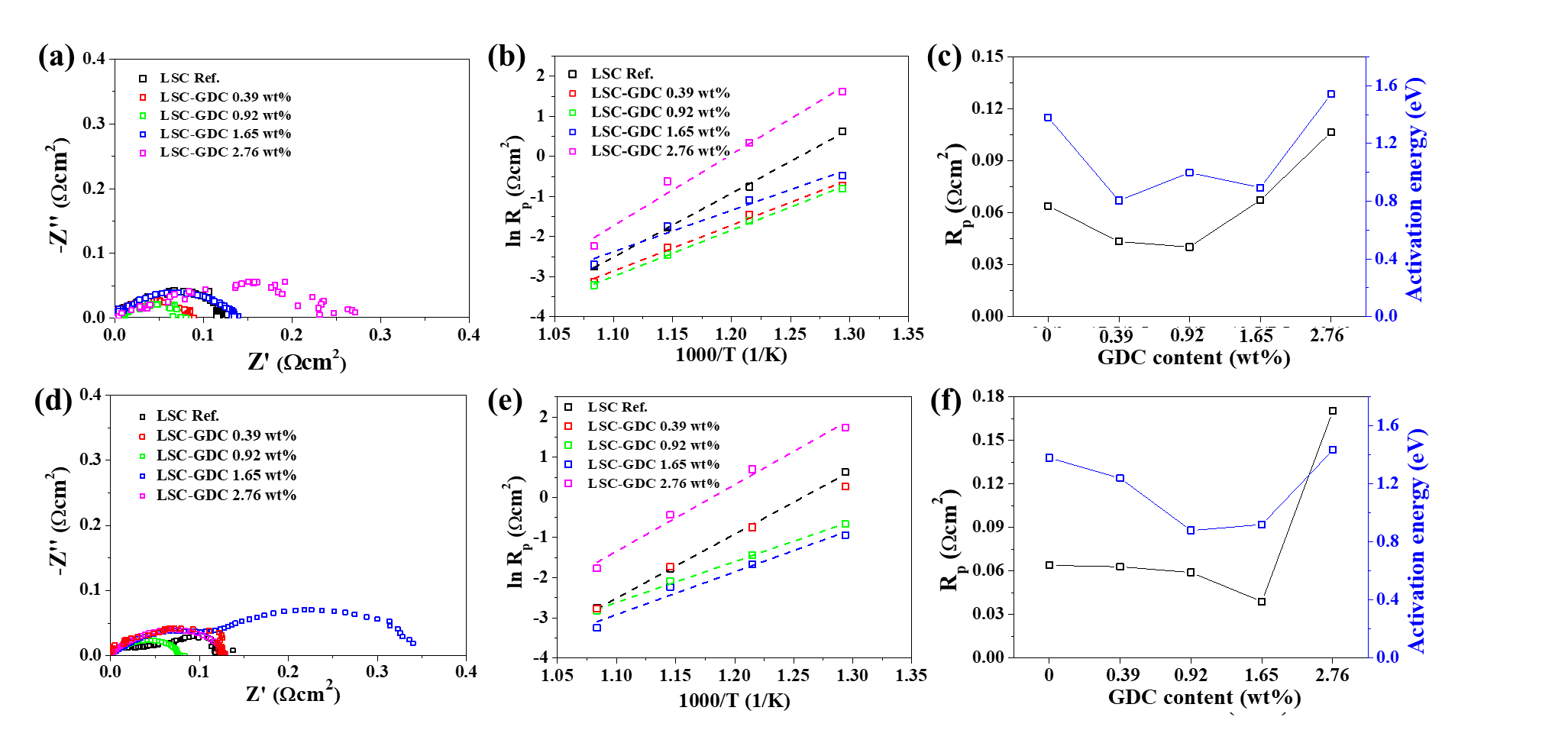


**Supplementary Figure 4.** Impedance spectra, Arrhenius plot, and R_p_ and activation energy with respect to the GDC content of LSC-GDC (a–c) with conventional ESD and (c–e) with SGC-assisted ESD, respectively

**
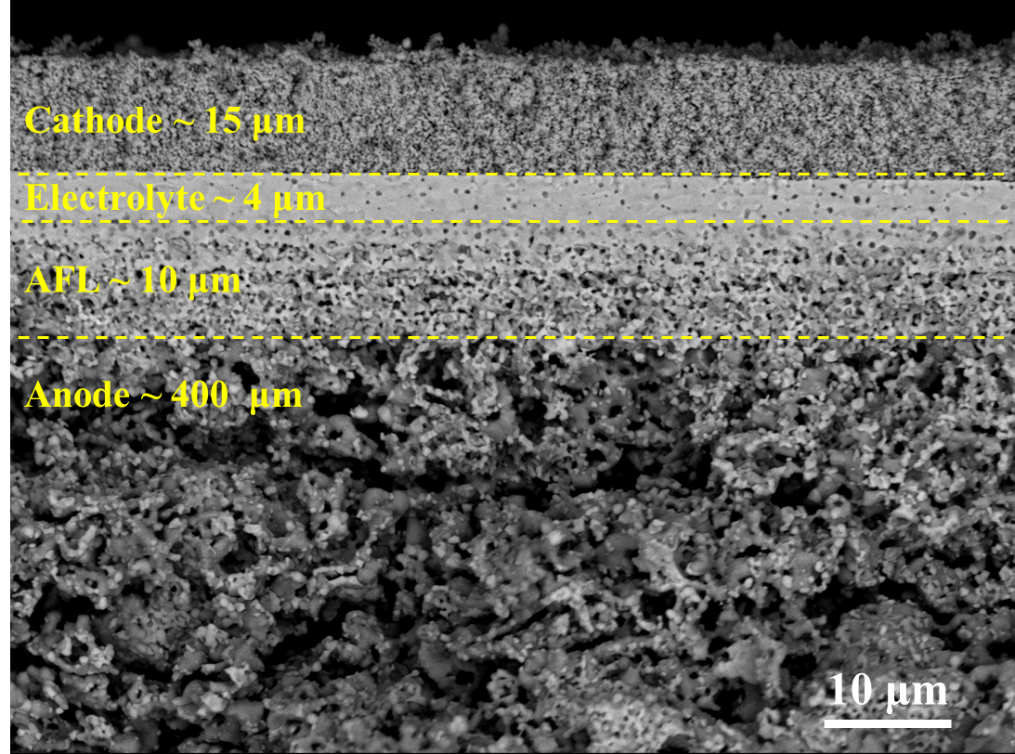
**

**Supplementary Figure 5.** SEM image of full cell cross-section

**Supplementary Figure 6.** Arrhenius plot of single cells with LSC electrode and LSC-GDC electrode fabricated by SGC-assisted ESD for the R_p_ and R_o_ and corresponding activation energies
